# The use of ultra-high frequency ultrasound in identifying aganglionosis in Hirschsprung’s disease

**DOI:** 10.1038/s41598-025-99897-7

**Published:** 2025-04-29

**Authors:** Tebin Hawez, Maria Evertsson, Tobias Erlöv, Kristine Hagelsteen, Louise Tofft, Tomas Jansson, Magnus Cinthio, Christina Granéli, Pernilla Stenström

**Affiliations:** 1 Department of Clinical Sciences Lund/Pediatrics, Lund University, Lund, Sweden; 2 Department of Clinical Sciences Lund/Biomedical Engineering, Lund University, Lund, Sweden; 3 Department of Biomedical Engineering, The Faculty of Engineering, Lund University, Lund, Sweden; 4 Department of Pediatric Surgery, Children’s Hospital, Skåne University Hospital, Lund, Sweden; 5 Digitalisering IT/MT, Skåne Regional Council, Lund, Sweden

**Keywords:** Paediatric research, Biomedical engineering, Motility disorders

## Abstract

To make surgery more precise and to shorten anesthesia-times for children undergoing surgery for Hirschsprung’s disease, ultra-high frequency ultrasound (UHFUS) has been suggested to replace peroperative biopsy. The study’s aim was to determine quantitatively whether aganglionic and ganglionic bowel segments could be distinguished by UHFUS. Bowel specimens of 23 children operated on for rectosigmoid Hirschsprung’s disease were examined ex vivo using the Vevo MD UHF70 (MHz) probe delivering 30 μm-resolution. In ganglionic versus aganglionic bowel in UHFUS images, patients serving their own controls, the thickness of the muscularis interna was thicker (0.540 vs. 0.322 mm; *p* < 0.001; CI 0.148–0.289), the ratio of thickness of muscularis interna/muscularis externa was greater (1.194 vs. 0.846; *p* = 0.011; CI 0.085–0.596), and the submucosa’s echogenicity was lower (100.0 vs. 115.3; *p* < 0.001; CI − 21.8 to − 8.7). The results indicate that delineation between aganglionosis and ganglionosis can be undertaken by UHFUS, underlining important research for precise diagnostics with UHFUS in vivo.

## Introduction

Hirschsprung’s disease is acknowledged by the European Council to be a rare disease^1^ occurring in approximately 1/5000 live births^2^. The disease is congenital and characterized by the absence of enteric ganglionic cells in bowel wall (aganglionosis), implying a life-threatening obstruction, requiring surgical resection^3^. The aganglionosis extends orally from the anus, involving the rectosigmoid colon in the majority of cases, but the exact length of the bowel affected depends on the individual, and fresh frozen biopsy diagnostics during surgery have been mandatory to detect ganglionic cells in the resection line^4^.

The clinical problem is that the waiting time for fresh frozen biopsy analyzes implies prolonged intraoperative anesthesia times, which become of special concern when multiple frozen biopsies are required. Other clinical problems with frozen biopsy are those connected to the approximability, i.e. that of the site of the frozen biopsy is decided only upon visual judgment of the surgeon, and to the insecurity of diagnostics since frozen biopsy does not allow proper histopathology staining. To address the diagnostic accuracy and surgical exactness, a more instant and precise method of delineating between ganglionic and aganglionic bowel is warranted. For this purpose, ultra-high frequency ultrasound (UHFUS) has been proposed^5^. UHFUS (20–70 MHz) provides detailed images of histoanatomic structures down to 30 μm, in superficial tissue depths. The clinical potential of UHFUS has been reported in the clinical fields of oral mucosal diagnostics^6^ and in neonatal abdominal wall diagnostics^7^.

In studies of the bowel wall, UHFUS imaging has been proven to reproduce histoanatomic differences between aganglionic and ganglionic bowel^8,9^. Therefore the point has been raised that there is a potential clinical use of UHFUS as a quick and safe diagnostic method in Hirschsprung’s disease, which could spare children complications and prolonged anesthesia times. The knowledge gap remains regarding whether histoanatomic differences between aganglionic and ganglionic bowel on UHFUS imaging can be proven quantitatively. To explore this, the aim of this study was to compare histoanatomic quantitative features of aganglionic and ganglionic bowel examined ex vivo, as imaged by UHFUS. The first research question was whether the histoanatomic thicknesses of the muscularis interna and muscularis externa differ between aganglionic and ganglionic bowel wall on UHFUS imaging (70 MHz), and if any difference of the visible presence of the myenteric interstitial layer can be identified. The second research question was whether the echogenicity (whiteness), in the UHFUS images of the muscularis interna, muscularis externa and submucosa, respectively, and the relative differences between them, differed between aganglionic and ganglionic bowel wall.

Based on our previous studies^5,8–10^ and clinical experiences by using UHFUS on the bowel wall, the hypothesis was the following: That the muscularis interna would be thicker in ganglionosis versus aganglionosis, that this would present with a relatively greater thickness of muscularis interna, and that the myenteric intestinal layer would more often be visible in ganglionosis. The echogenicity of the muscularis interna and submucosa was hypothesised to be higher in aganglionic versus ganglionic segments.

## Materials and methods

### Settings

This was an observational interventional study carried out at a Department of Pediatric Surgery, appointed by the country’s healthcare authorities to be a national referral center for Hirschsprung’s disease from 2018. The diagnosis of Hirschsprung’ s disease followed a continuously updated local diagnostic guideline, based on an algorithm including symptoms, radiologic evaluation with a colonic contrast enema, and mandatory rectal or colonic biopsy with histopathological and immunohistochemical staining. All children diagnosed with Hirschsprung’s disease and scheduled to undergo surgical resection of the aganglionic segment between April 2018 and August 2022 were eligible for inclusion. Inclusion criteria were rectosigmoid aganglionosis stretching between 10 and 30 cm orally from anus, in children weighing a maximum of 10 kg and of ages younger than 1 year at the time of surgery. Information about the participants included the patient’s age, weight, and resected bowel length after formalin- and paraffin embedding, and was retrieved from the local and prospectively reported register held for Hirschsprung’s disease.

### The research procedure, technical settings and specimen treatment

**Fig. 1:**
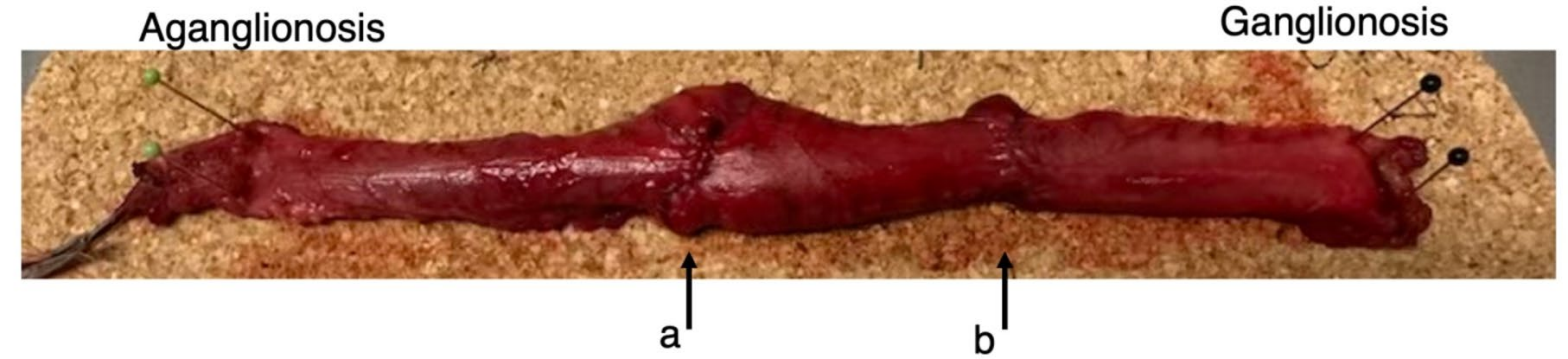
The resected bowel was pinned to a cork mat and examined in full double layer position with ultra-high frequency ultrasound. Aganglionosis and ganglionosis were verified through histological and immunohistochemistry staining. The arrows indicate sites for frozen biopsy taken during surgery; a with absence of ganglia cells and b showing the transition zone.

The surgical resection length was decided upon intraoperatively by taking fresh frozen biopsies of the bowel wall at any level, as preferred by the surgeon, confirming ganglionic bowel wall. The resected bowel wall segment was pinned to a cork mat and examined with UHFUS by two pediatric surgeons from the serosa surface at sites representing ganglionosis and aganglionosis (Fig. 1). Gel was used as a conductor. All specimens were examined with UHFUS by researchers, collecting longitudinal views of the bowel wall. The Vevo MD Ultra High Frequency Ultrasound imaging system (Fujifilm VisualSonics, Toronto, ON, Canada) with an UHF70 linear-array transducer (bandwidth 29–71 MHz, center frequency 50 MHz) was used. The UHFUS imaging in this study followed a predefined acquisition protocol of 50 MHz center frequency, 100% power, 48 dB gain, maximum 7 mm imaging depth, 9.73 mm width, persistence off and 65 dB dynamic range. The bowel wall visualisation was saved as a two-dimensional ultrasound image (also known as a B-mode image). For each patient, two UHFUS images were obtained: one at the ganglionic site and one at the aganglionic site. Ultrasound image data were saved and exported for further analysis using an in-house developed software program^10^. During the UHFUS examination, the proposed aganglionic and ganglionic parts of the bowel were indicated by pins. These were used during assessment by the pathologist to confirm or reject the presence of aganglionosis. After UHFUS imaging, the resected specimen was fixed in formalin, embedded in paraffin, and processed histopathologically and immunohistologically with hematoxylin and eosin, calretinin and S-100, respectively, according to the state-of-the-art diagnostics in Hirschsprung’s disease^11–13^. The resected specimen was evaluated by subspecialized pediatric clinical pathologists to confirm or reject the presence of aganglionic and ganglionic segments at the depicted sites of UHFUS imaging.

### Assessment of UHFUS images

UHFUS images were exported for assessment in semi-automatic in-house software program, based on MATLAB (MathWorks Inc, Natick, MA, USA). The program was developed specifically for the bowel wall and has been validated between two observers^10^. A 5-mm wide region of interest (ROI), covering the best image quality, was chosen for each image. Within the ROI, the outer and inner borders of the muscularis externa, muscularis interna and the myenteric tissue layer between the muscularis layers, were delineated manually. For the submucosa, only the outer border was delineated since the inner border was difficult to visualize as a result of the limited depth penetration of UHFUS. Instead, a fixed predetermined depth of 0.18 mm was used to define a region of the submucosa layer.

After the manual delineation of the histoanatomic layers of the inner and outer border of the muscularis interna and externa, respectively, quantitative measures of thicknesses and echogenicity were generated automatically by the software program. The thickness, measured for the muscularis externa and the muscularis interna, was defined as the mean of several consecutive thicknesses (measured in lateral steps of 32 μm). The myenteric tissue layer was reported as identified or not (1/0). Echogenicities were defined as the mean echogenicity within the muscularis interna, muscularis externa and submucosa, respectively.

### Statistics

Thicknesses (millimeters) and echogenicity (arbitrary units) of histoanatomic layers in bowel wall were presented as mean (standard deviation; SD) and differences displayed by confidence intervals (CI). For relative differences between the histoanatomic muscular layers, ratios of thicknesses of the muscularis interna/muscularis externa were calculated. Also the echogenicity, and ratios of the echogenicity of the muscularis interna/muscularis externa, muscularis interna/submucosa and muscularis externa/submucosa were calculated. These calculations were made in Microsoft Excel, and IBM SPSS Statistics (v.27) was used for statistics.

Statistical methods and quality control were performed by the Department of Statistics at the hospital. Distribution patterns of differences in thicknesses and echogenicity were tested in histograms, presenting symmetric distribution, indicating use of parametric statistical tests. Each patient served as their own control so differences between aganglionic and ganglionic bowel were analyzed using the paired t-test and CI. For the myenteric tissue layer, dichotomization identification (yes/no) was analyzed with McNemar’s test (0 values present). A two-sided p-value < 0.05 was considered to be statistically significant.

### Ethical considerations

Ethical approval was obtained from the Local Ethical Review Board (DNR 2017/769) and the Swedish Ethical Review Authority (DNR 2023-01833-01). The research was performed in accordance with regional and national guidelines and regulations. Informed consent was obtained from all the participants and/or their legal guardians.

## Results

**Fig. 2:**
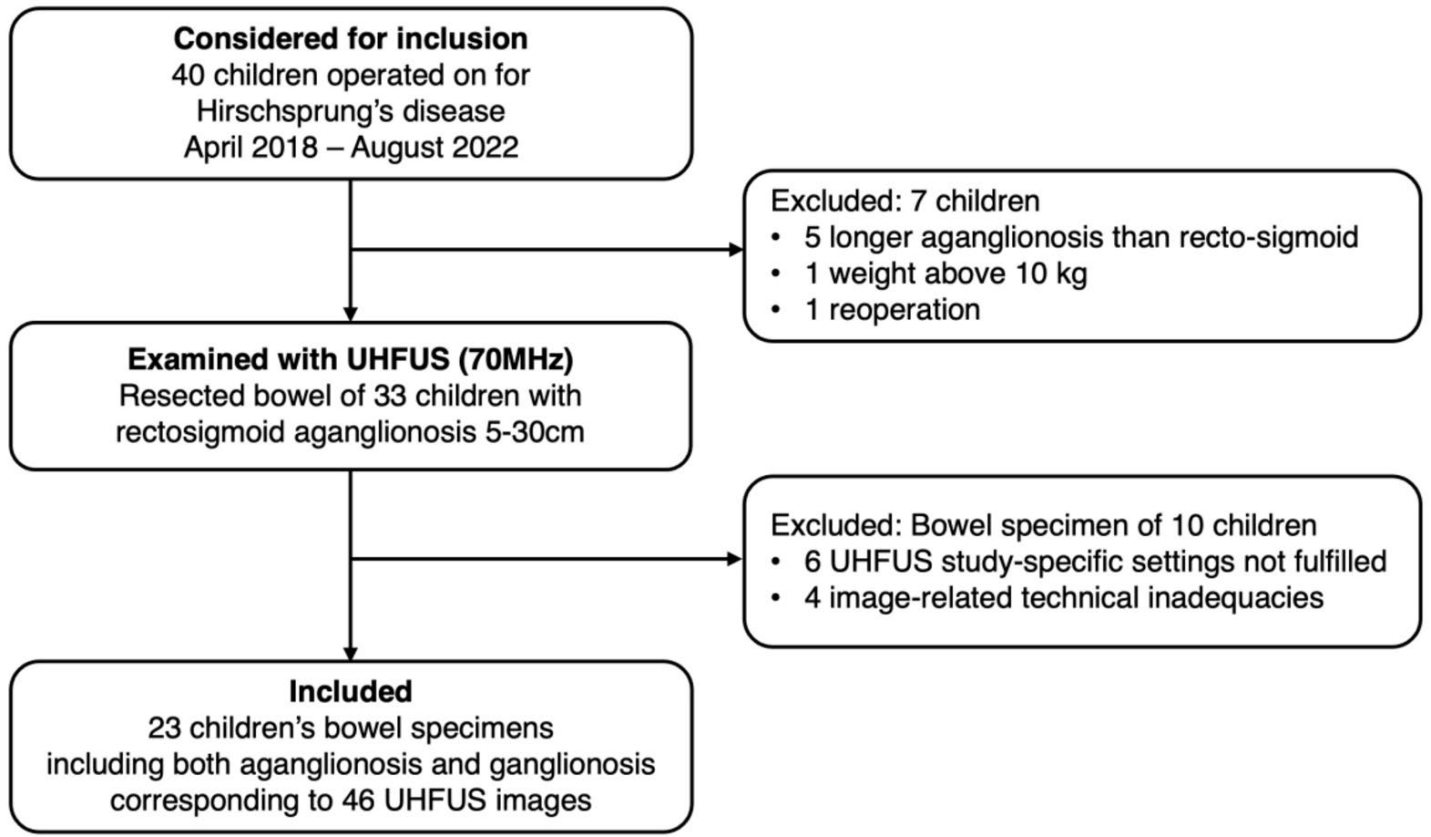
Flow chart of study inclusion in the study on resected bowel segments of children with rectosigmoid Hirschsprung’s disease, examined ex vivo with ultra-high frequency ultrasound (UHFUS).

During the study period, a total of 40 children underwent surgery consecutively for Hirschsprung’s disease. After exclusions (aganglionosis stretching longer than 30 cm *n* = 5, weight above 10 kg *n* = 1, reoperation *n* = 1, non-arterial pre-set of the UHFUS *n* = 4, missing ultrasound data *n* = 6), resected rectosigmoid bowel with aganglionic and ganglionic segments of 23 children were included (Fig. 2). The median age and weight of the included patients at the time of surgery were 34 days (range 11–174 days) and 4100 g (range 2600–7700 g), respectively, and the median length of the resected aganglionic bowel wall was 19 cm (range 7–26 cm).

**Fig. 3:**
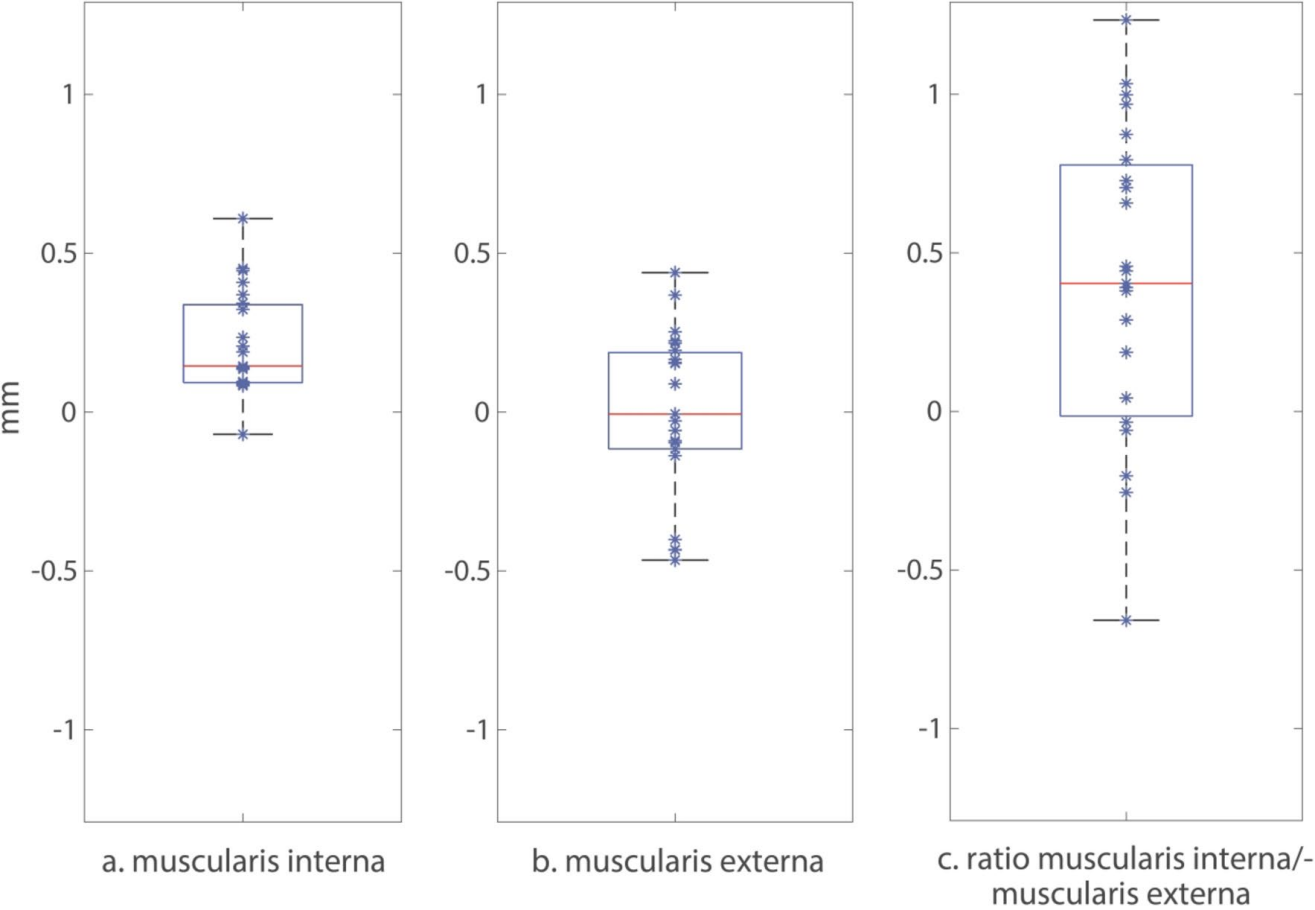
**(a–c)** Boxplots of differences in histoanatomic thickness (**a,b**) and ratio of thicknesses (**c**) between ganglionic and aganglionic (ganglionosis minus aganglionosis) bowel wall imaged ex vivo with ultra-high frequency ultrasound in 23 children operated on for recto-sigmoid Hirschsprung’s disease. Each dot represents one patient’s difference. From left to right: (**a**) thickness difference in muscularis interna, (**b**) thickness difference in muscularis externa and (**c**) thickness ratio difference for muscularis interna/muscularis externa.

**Fig. 4:**
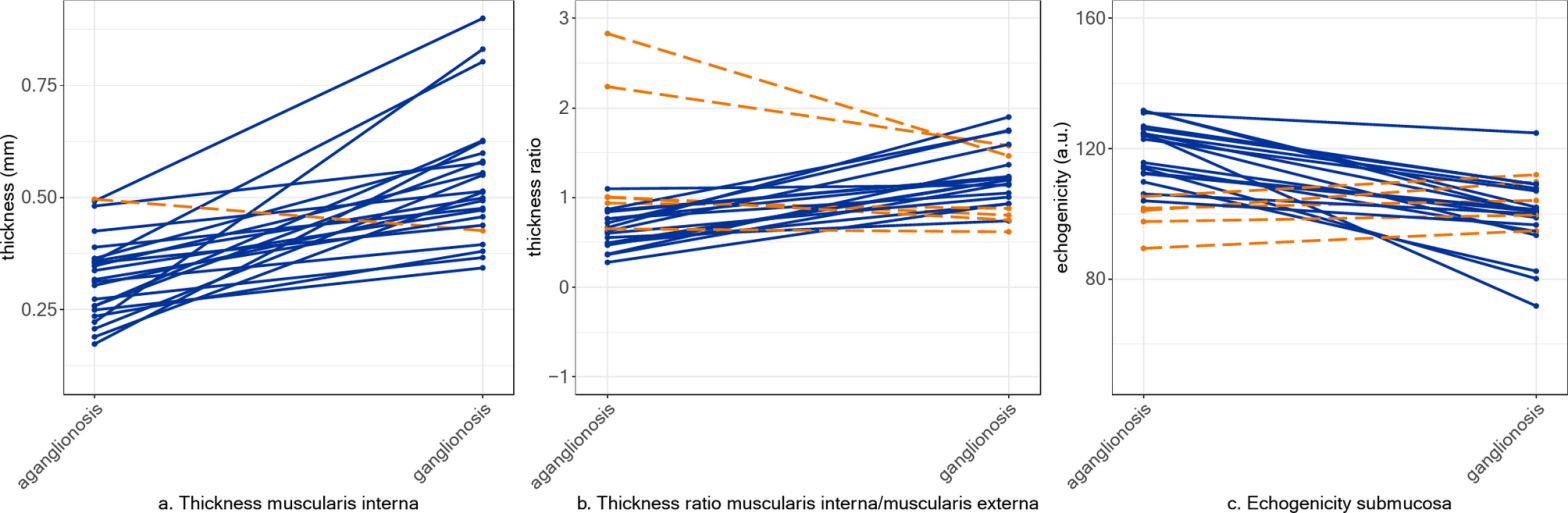
**(a–c)** Spaghetti plots visualizing differences in thicknesses (**a,b**) and image echogenicity (**c**) between aganglionosis (left) and ganglionosis (right) in each patient. Figure (**a**) thickness of muscularis interna, (**b**) thickness ratio of muscularis interna/muscularis externa and (**c**) image echogenicity (arbitrary units; a.u.) of the submucosa. Blue lines indicate greater thicknesses in ganglionosis and lower echogenicity in ganglionosis, while dotted orange lines indicate the opposite, in each unique bowel segment (*n* = 23).

Overall, the mean muscularis interna thickness was greater in ganglionic bowel (0.540 mm) compared to aganglionic bowel (0.322 mm) being 82% thicker in ganglionic bowel (*p* < 0.001). The thickness of the muscularis externa did not differ between aganglionosis and ganglionosis. The ratio of the muscularis interna/muscularis externa was 81% greater in ganglionosis (*p* = 0.011) (Table 1). The muscularis interna was thicker in ganglionosis than in aganglionosis in 96% (22 out of 23) specimens (Fig. 3a) and these differences in thickness presented with a great consistency (Fig. 4a). The thickness of the muscularis externa differed widely and without consistency (Fig. 3b). In the single specimen, in which the muscularis interna was not thicker in ganglionosis, the ratio of the muscularis interna/muscularis externa was still greater (Fig. 4a). Overall, the ratio of the muscularis interna/muscularis externa was greater in ganglionosis (Fig. 3c), where a higher ratio was present in 74% (17 out of 23) specimens (Fig. 4b).

**Table 1:** Differences in histoanatomic thicknesses in bowel wall imaged ex vivo with ultra-high frequency ultrasound comparing aganglionic and ganglionic segments in 23 children operated on for recto-sigmoid Hirschsprung’s disease, with patients serving as their own controls.

	Aganglionosis Mean (SD)	Ganglionosis Mean (SD)	Percentage difference (%) between aganglionosis and ganglionosis Mean (SD)	Real difference Mean (CI)*	*p*-value*	Patients with greater value in ganglionosis *n* (%)
Muscularis interna (mm) *n* = 23	0.322 (0.094)	0.540 (0.145)	82.2 (76.6)	0.218 (0.148, 0.289)	**< 0.001**	22 (96)
Muscularis externa (mm) *n* = 23	0.476 (0.224)	0.478 (0.140)	23.5 (66.6)	0.007 (− 0.104, 0.118)	0.896	11 (49)
Muscularis interna/muscularis externa *n* = 23	0.846 (0.583)	1.194 (0.351)	81.0 (92.8)	0.340 (0.085, 0.596)	**0.011**	17 (74)

*Paired samples t test, *p* < 0.05 was considered statistically significant.

Confidence interval 95% (CI). Standard deviation (SD). Percentage (%).

A myenteric tissue layer was identifiable in 52% (12 out of 23) ganglionic segments compared to 13% (3 out of 23) in aganglionic segments (*p* = 0.012). In two specimens a myenteric layer was identified in both the ganglionic and aganglionic segments, while in 10 specimens a myenteric layer was not visible in neither ganglionosis nor aganglionosis (Table 2).

**Table 2:** Difference of visualized myenteric tissue layer, as visualized on ultra-high frequency ultrasound, comparing the histoanatomic layer’s presence on images in aganglionic and ganglionic segments, respectively, in 23 children operated on for recto-sigmoid Hirschsprung’s disease.

		Ganglionosis *n* = 23			p-value*
		Visible (n)	Not visible (n)	Total (n)	
Aganglionosis *n* = 23	Visible (n)	2	1	3	0.012
	Not visible (n)	10	10	20	
	Total (n)	12	11	23	

* McNemar’s test, *p* < 0.05 was considered statistically significant.

The histoanatomic layer with the greatest difference in echogenicity comparing ganglionic and aganglionic bowel was the submucosa. The echogenicity of the submucosa was lower (darker) in ganglionic segments, differing by a mean of 12% from aganglionic segments (*p* < 0.001) (Table 3) and being lower in ganglionosis in 78% of the specimens (18 out of 23) (Fig. 4c). The echogenicity of the muscularis interna was overall also lower in ganglionic bowel wall, being 8.7% lower in ganglionosis (*p* = 0.009), and being lower in 78% of the specimens (18 out of 23). The echogenicity of the muscularis externa did not differ, although in 17 specimens it was lower in ganglionosis. Comparing relative differences of echogenicities, i.e. ratios, the echogenicity ratio of the muscularis externa/submucosa was greater in ganglionosis versus aganglionosis (*p* = 0.015) while the ratios of the muscularis interna/muscularis externa and the muscularis interna/submucosa, respectively, did not differ between ganglionosis and aganglionosis (Table 3).

**Table 3:** Differences in image echogenicity of histoanatomic layers in bowel wall comparing aganglionosis and ganglionosis segments imaged ex vivo with ultra-high frequency ultrasound in 23 children operated on for recto-sigmoid Hirschsprung’s disease, with patients serving as their own controls.

	Aganglionosis Mean (SD)	Ganglionosis Mean (SD)	Mean real difference (CI)*	Percentage difference (%) between aganglionosis and ganglionosis Mean (SD)	*p*-value*	Patients with greater value in aganglionosis *n* (%)
Muscularis interna *n* = 23	91.4 (13.0)	82.2 (12.5)	− 9.2 (− 15.8, − 2.6)	− 8.7 (16.9)	**0.009**	18 (78)
Muscularis externa *n* = 23	121.1 (13.7)	115.3 (10.9)	− 5.7 (− 12.2, − 0.8)	− 3.8 (13.0)	0.083	17 (74)
Submucosa *n* = 23	115.3 (12.0)	100.0 (11.2)	− 15.3 (− 21.8, − 8.7)	− 12.4 (12.7)	**< 0.001**	18 (78)
Muscularis externa/ submucosa *n* = 23	1.059 (0.148)	1.162 (0.131)	0.1 (0.0, 0.2)	11.6 (19.3)	**0.015**	7 (30)

*Paired samples t test, *p* < 0.05 was considered statistically significant.

Confidence interval 95% (CI). Standard deviation (SD). Percentage (%).

**Fig. 5:**
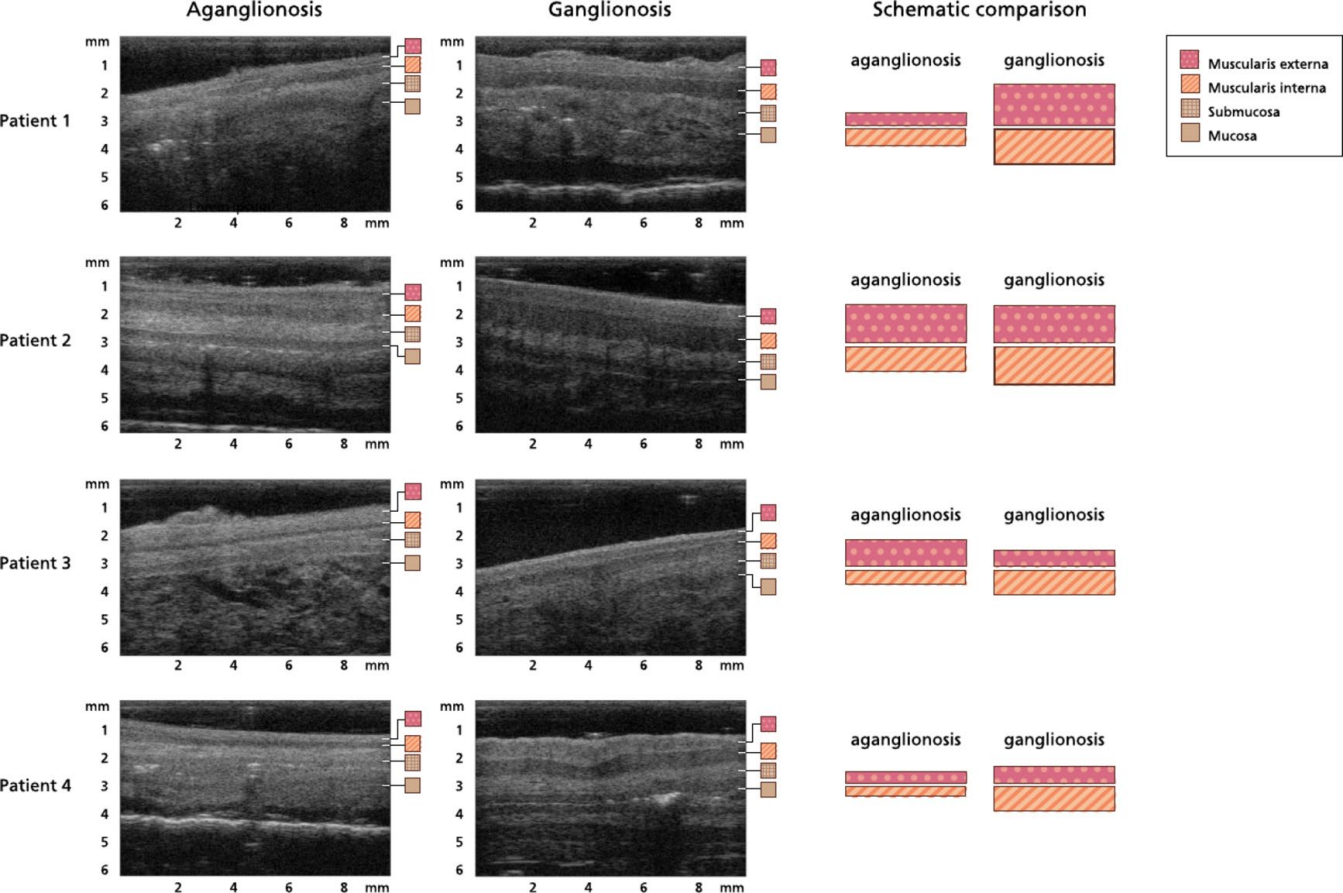
Ultra-high frequency ultrasound images of aganglionic and ganglionic bowel wall segments in patients operated on for recto-sigmoid Hirschsprung’s disease. In each image, different bowel wall layers are represented by different patterned squares. To the right, each patient’s aganglionic and ganglionic thicknesses of muscularis externa and interna are presented graphically side by side. In the ganglionic bowel wall images of patients 1,3 and 4, a myenteric tissue layer between the muscularis externa and muscularis interna is identifiable.

An overview of typical images of the histoanatomic thicknesses and echogenicities in aganglionosis and ganglionosis is displayed in Fig. 5.

## Discussion

The study’s results revealed that histoanatomic differences between aganglionic and ganglionic bowel wall could be identified clearly with UHFUS in bowel examined ex vivo. Specifically, we found that the muscularis interna was thicker and the ratio of the muscularis interna/muscularis externa was greater in ganglionic compared to aganglionic bowel, reflecting a consistent difference. The echogenicity of both the submucosa and the muscularis interna was lower in ganglionosis than in aganglionosis. All together, these findings suggest strongly that UHFUS is extremely useful for delineation of aganglionosis during Hirschsprung’s disease surgery and potentially can reduce the amount of time children have to be anesthetised in order that fresh frozen biopsies can be taken during surgery.

Ultrasound diagnostics of bowel wall has only been explored to a limited extent before, and exclusively within a standard clinical frequency range (8.5-25Mz), concluding a need for more detailed histoanatomic visualisation in clinical use^14,15^. In contrast to such standard clinical frequency ultrasound, UHFUS (70 MHz) has been shown to reproduce the histoanatomy of bowel wall very well and in detail^8^. In oral epithelium, UHFUS has been reported to enable visualisation of details and delineation between malignant lesions and healthy oral tissue^16^. These findings formed the basis to our hypothesis that UHFUS could also be useful in diagnostics of the bowel wall. However, in previous studies, quantified measures of UHFUS images were lacking. Therefore, the present study’s novel data on quantifications of histoanatomy on UHFUS fill the lack of knowledge about the bowel wall.

Regarding histoanatomic thicknesses on UHFUS, our results are supported by those of our previous study on paraffin embedded and histopathologically stained specimens in which histoanatomic metrics showed a thicker muscularis interna and a greater ratio of muscularis interna/muscularis externa in ganglionic bowel^9^. These findings are in line with results of those of murine intestinal obstructive models^17,18^. In our study, the UHFUS replication showed that some specimens presented with both the muscle layers of the bowel wall being thicker in ganglionosis. Still, reflected in a larger ratio of the muscularis interna/muscularis externa, in these specimens the thickness of the muscularis interna was relatively greater than that of the externa. An important result in our study was that the thickness of the muscularis externa, in contrast to that of the muscularis interna, varied greatly in both aganglionic and ganglionic bowel wall. The reason that the muscularis interna and muscularis externa seem to react differently on aganglionosis or obstruction has not yet been clarified.

Regarding the echogenicity, it was particularly higher in the submucosa of aganglionic bowel. However, the muscularis interna also presented with higher echogenicity in aganglionosis than in ganglionosis. Higher echogenicity (whiter color) is known to be evident in more dense tissue, as, for example, when collagen is present^19^. In Hirschsprung’s disease, a higher image echogenicity on UHFUS could, hypothetically, be the result of a higher content of collagen fibres secondary to a lack of ganglionic cells, or due to nerve fascicle hypertrophy in aganglionosis^20^. This could, speculatively, be the case in the submucosa where ganglionic cells are usually present but might be replaced by collagen in aganglionosis. On the other hand, the myenteric tissue layer, also constituting an important histoanatomic site for ganglionic cells, was visible more frequently in ganglionic bowel wall. This is a result in line with our previous histopathology studies^9^. Reasons for a more prominent appearance of the myenteric layer on UHFUS could speculatively be due to a dense presence of ganglionic cells in ganglionosis, because the UHFUS visualizes sizes down to 30 μm which is the size of the myenteric nerve cells^9^. Or it could be secondary to a contrasting effect of the surrounding echogenicity, being lower in ganglionosis. However, since the myenteric tissue layer varied considerably in its presence between patients, we consider the role of it in UHFUS diagnostics of Hirschsprung’s disease to be weak, an observation remaining to be explored further.

The most important uncertainty for using UHFUS in Hirschsprung’s disease diagnostics is whether differences between aganglionic and ganglionic bowel wall really are disease-dependent or if they reflect a normal alternation between rectal and colonic bowel wall. In studies of humans and cats without significant bowel pathology, increased thickness of the muscularis propria (muscularis interna and muscularis externa together) in the aboral direction has been suggested^21,22^. To confirm any disease-specific histoanatomic differences, a case-control study of healthy individuals versus Hirschsprung’s disease patients would be required. Since very few children of neonatal age without gastrointestinal malformations are operated upon, such case-control studies constitute an ethical and practical problem. Therefore alternative models to find out whether the thickness of the muscularis interna alters normally are being explored, using modalities not requiring surgery.

A strength of this study was the homogeneity of the patient cohort, having similar ages, weights and exclusively representing rectosigmoid Hirschsprung’s disease. Moreover, since patients could serve as their own controls when conducting statistics, the effects of confounding factors were minimized. Detailed manual delineations as well as automatic calculations could be used as a result of the software program designed specifically for this project. The program and calculations have been proven to present with a high agreement between users in interpersonal validation^10^. The study is exploratory, and its novelty so high that there are no similar studies on UHFUS on human bowel wall to compare with it. Therefore, all the here-presented results will need to be replicated, confirmed or rejected in larger multicenter studies, and especially in in vivo settings, in order to achieve clinical usefulness. In particular, the clinical implementation for total colonic ganglionosis is expected to take longer, as the group is heterogeneous and the number of patients is limited.

A limitation of the study is the limited number of specimens included in the analyzes, reflecting the rarity of Hirschsprung’s disease. Although every consecutive child diagnosed with Hirschsprung’s disease at the center was considered for inclusion, the number within the pre-set study time frame ended up being limited, increasing the probability of a type II error. This size limitation is not only recognized in studies on rare diseases, but also in studies on novel technologies, of which both are represented in this study. In order to include more patients, transporting the UHFUS technology to other pediatric surgical centers, or supplying finances to other sites so that they can also buy the UHFUS machine for study purposes, need to be covered. Also, an unconscious selection bias cannot be ruled out, as approximately 30% of the patients undergoing surgery were excluded either for not meeting the study-specific requirements or due to imaging-related technical issues. Another important limitation to address is that a part of the examinations used in this study have been reported on in our previous studies on histopathological correlation and validation of the software^8,10^. Another limitation regards the potential examination bias by the examiner’s exerted pressure with the transducer^23^ on bowel wall and/or position of the transducer on the bowel. Although care was taken not to exert pressure on the specimen, and the gel was confirmed in all images, a certain tissue pressure cannot be excluded implicating on histoanatomic thicknesses and image echogenicity. Also, echogenicity depends on the ultrasound imaging system used, the type of transducer, and transducer handling. Also, ultrasound has been shown to be highly operator dependent^24^. Therefore any reproducibility is most likely achieved by using ratios, as used, rather than real thicknesses or echogeniticities of single layers. Ratios will probably be of especial importance in in vivo examinations when the same background material or tissue cannot be guaranteed. Another concern could be potential variations in imaging depending on the child’s age or weight. However, examining any correlation between age and bowel wall thickness was not feasible with the UHFUS 70, as its depth did not fully cover the entire bowel wall.

Looking ahead, the UHFUS method on bowel wall is very encouraging. The here-presented study is part of an ongoing larger multidisciplinary project aiming to validate UHFUS as a diagnostic method. Future clinical implications may include support of both intraoperative delineation of aganglionic bowel in vivo as well as primary diagnostics of Hirschsprung’s disease by anal examinations^25^.

In the development of UHFUS for clinical use, ongoing studies are being conducted to examine bowel wall characteristics in aganglionosis, the transition zone, and ganglionosis. In ongoing studies the use of UHFUS in assessment of the transition zone is specifically focused. The here-presented results form an important basis for all these ongoing and future studies. Importantly, all diagnostics of Hirschsprung’s disease still need histopathological confirmation or rejection.

## Conclusion

This study reveals significant quantitative differences in both real and relative histoanatomic thicknesses and echogenicities between aganglionic and ganglionic bowel wall of rectosigmoid Hirschsprung’s disease visualized by UHFUS. The increased thickness of the muscularis interna and the reduced image echogenicities of the muscularis interna and submucosa in ganglionosis are highly valuable for UHFUS- and algorithm-based diagnostics. Our findings contribute to more effective, safe, and precise disease detection in children with Hirschsprung’s disease.

## Data Availability

Materials requests and correspondence should be addressed to Tebin Hawez.

## References

[1] European Commission. *European Reference Networks*. https://ec.europa.eu/health/sites/health/files/ern/docs/2017_brochure_en.pdf (Accessed 15 November 2024) (2017).

[2] Löf Granström, A. et al. Maternal risk factors and perinatal characteristics for hirschsprung disease. *Pediatrics* **138**, 8. 10.1542/peds.2015-4608 (2016). 10.1542/peds.2015-4608 27307146

[3] Langer, J. C. Hirschsprung disease. *Curr. Opin. Pediatr.* **25**, 368–374. 10.1097/MOP.0b013e328360c2a0 (2013). 23615177 10.1097/MOP.0b013e328360c2a0

[4] Neuvonen, M. I. et al. A population-based, complete follow-up of 146 consecutive patients after Transanal mucosectomy for hirschsprung disease. *J. Pediatr. Surg.* **50**, 1653–1658. 10.1016/j.jpedsurg.2015.02.006 (2015). 25783387 10.1016/j.jpedsurg.2015.02.006

[5] Granéli, C. et al. Ultra high frequency ultrasonography to distinguish ganglionic from aganglionic bowel wall in hirschsprung disease: A first report. *J. Pediatr. Surg.* **56**, 2281–2285. 10.1016/j.jpedsurg.2021.02.011 (2021). 33676743 10.1016/j.jpedsurg.2021.02.011

[6] Izzetti, R. et al. Ultra-high frequency ultrasound, a promising diagnostic technique: review of the literature and single-center experience. *Can. Assoc. Radiol. J.* **72**, 418–431. 10.1177/0846537120940684 (2021). 32721173 10.1177/0846537120940684

[7] Jacobsen, R. B., Hebelka, H., Gatzinsky, V., Elfvin, A. & Dangardt, F. Ultra-high-frequency ultrasound (48–70 MHz) is a promising tool for improved Gastrointestinal diagnostics in infants. *Acta Paediatr.* **1**, 1. 10.1111/apa.17342 (2024). 10.1111/apa.17342 38953873

[8] Hawez, T. et al. Ultra-high frequency ultrasound imaging of bowel wall in Hirschsprung’s disease-correlation and agreement analyses of histoanatomy. *Diagnostics (Basel)*. **13**, 88. 10.3390/diagnostics13081388 (2023). 10.3390/diagnostics13081388 PMC10137420 37189490

[9] Graneli, C. et al. Histopathological dimensions differ between aganglionic and ganglionic bowel wall in children with Hirschsprung’s disease. *BMC Pediatr.* **22**, 723. 10.1186/s12887-022-03792-3 (2022). 36536313 10.1186/s12887-022-03792-3 PMC9764572

[10] Erlöv, T. et al. A computer program for assessing histoanatomical morphometrics in ultra-high-frequency ultrasound images of the bowel wall in children: development and inter-observer variability. *Diagnostics (Basel)*. **13**, 59. 10.3390/diagnostics13172759 (2023). 10.3390/diagnostics13172759 PMC10486739 37685297

[11] Bachmann, L. et al. Immunohistochemical panel for the diagnosis of Hirschsprung’s disease using antibodies to MAP2, calretinin, GLUT1 and S100. *Histopathology* **66**, 824–835. 10.1111/his.12527 (2015). 25123159 10.1111/his.12527

[12] Takawira, C., D’Agostini, S., Shenouda, S., Persad, R. & Sergi, C. Laboratory procedures update on hirschsprung disease. *J. Pediatr. Gastroenterol. Nutr.* **60**, 598–605. 10.1097/mpg.0000000000000679 (2015). 25564805 10.1097/MPG.0000000000000679

[13] Galazka, P., Szylberg, L., Bodnar, M., Styczynski, J. & Marszalek, A. Diagnostic algorithm in Hirschsprung’s disease: focus on immunohistochemistry markers. *In Vivo* **34**, 1355–1359. 10.21873/invivo.11913 (2020). 32354930 10.21873/invivo.11913 PMC7279842

[14] Kimmey, M. B. et al. Histologic correlates of Gastrointestinal ultrasound images. *Gastroenterology* **96**, 433–441. 10.1016/0016-5085(89)91568-0 (1989). 2642877 10.1016/0016-5085(89)91568-0

[15] Wiersema, M. J. & Wiersema, L. M. High-resolution 25-megahertz ultrasonography of the Gastrointestinal wall: histologic correlates. *Gastrointest. Endosc*. **39**, 499–504. 10.1016/s0016-5107(93)70159-5 (1993). 8365596 10.1016/s0016-5107(93)70159-5

[16] Izzetti, R. et al. The efficacy of ultra-high frequency ultrasonography in the diagnosis of intraoral lesions. *Oral Surg. Oral Med. Oral Pathol. Oral Radiol.* **129**, 401–410. 10.1016/j.oooo.2019.09.012 (2020). 32009004 10.1016/j.oooo.2019.09.012

[17] Hillemeier, C. & Biancani, P. Mechanical properties of obstructed colon in a Hirschsprung’s model. *Gastroenterology* **99**, 995–1000. 10.1016/0016-5085(90)90618-b (1990). 2394354 10.1016/0016-5085(90)90618-b

[18] Gabella, G. Hypertrophy of intestinal smooth muscle. *Cell. Tissue Res.* **163**, 199–214 (1975). 1182787

[19] Levy, J. et al. High-frequency ultrasound in clinical dermatology: a review. *Ultrasound J.* **13**, 24. 10.1186/s13089-021-00222-w (2021). 33877462 10.1186/s13089-021-00222-w PMC8058126

[20] Wedel, T., Holschneider, A. M. & Krammer, H. J. Ultrastructural features of nerve fascicles and basal lamina abnormalities in Hirschsprung’s disease. *Eur. J. Pediatr. Surg.* **9**, 75–82. 10.1055/s-2008-1072217 (1999). 10342113 10.1055/s-2008-1072217

[21] Salvatierra, E. et al. W1445: regional differences in colon circumference and wall thickness. *Gastrointest. Endosc.* **71**, 58. 10.1016/j.gie.2010.03.858 (2010).

[22] Di Donato, P., Penninck, D., Pietra, M., Cipone, M. & Diana, A. Ultrasonographic measurement of the relative thickness of intestinal wall layers in clinically healthy cats. *J. Feline Med. Surg.* **16**, 333–339. 10.1177/1098612x13509080 (2014). 24174500 10.1177/1098612X13509080 PMC11383109

[23] Ihnatsenka, B., Boezaart, A. P. & Ultrasound Basic Understanding and learning the Language. *Int. J. Shoulder Surg.* **4**, 55–62. 10.4103/0973-6042.76960 (2010). 21472065 10.4103/0973-6042.76960 PMC3063344

[24] Farina, R. & Sparano, A. Errors in sonography. In *Errors in Radiology* (eds Romano, L. & Pinto, A.) (Springer, 2012).

[25] Evertsson, M. et al. Design of a pediatric rectal ultrasound probe intended for ultra-high frequency ultrasound diagnostics. *Diagnostics (Basel)*. **13**, 67. 10.3390/diagnostics13101667 (2023). 10.3390/diagnostics13101667 PMC10217470 37238152

